# Limitations of COVID-19 testing and case data for evidence-informed health policy and practice

**DOI:** 10.1186/s12961-023-00963-1

**Published:** 2023-01-25

**Authors:** Elizabeth Alvarez, Iwona A. Bielska, Stephanie Hopkins, Ahmed A. Belal, Donna M. Goldstein, Jean Slick, Sureka Pavalagantharajah, Anna Wynfield, Shruthi Dakey, Marie-Carmel Gedeon, Edris Alam, Katrina Bouzanis

**Affiliations:** 1 Department of Health Research Methods, Evidence and Impact, McMaster University, CRL 2nd Floor, 1280 Main Street West, Hamilton, ON L8S4K1 Canada; 2 Department of Anthropology, University of Colorado Boulder, Boulder, CO USA; 3 Disaster and Emergency Management, Royal Roads University, British Columbia, Canada; 4 School of Medicine, McMaster University, Hamilton, ON Canada; 5 Department of Architecture and Planning, Visvesvaraya National Institute of Technology, Nagpur, India; 6 University Integrated Health Center of the Nord-de-l’île de Montréal (CIUSSS NIM), Montréal, QC Canada; 7 Faculty of Resilience, Rabdan Academy, Abu Dhabi, United Arab Emirates; 8 Department of Global Health, McMaster University, Hamilton, ON Canada

**Keywords:** COVID-19, Testing, Epidemiology, Case count, Physical distancing policy

## Abstract

**Background:**

Coronavirus disease 2019 (COVID-19) became a pandemic within a matter of months. Analysing the first year of the pandemic, data and surveillance gaps have subsequently surfaced. Yet, policy decisions and public trust in their country’s strategies in combating COVID-19 rely on case numbers, death numbers and other unfamiliar metrics. There are many limitations on COVID-19 case counts internationally, which make cross-country comparisons of raw data and policy responses difficult.

**Purpose and conclusions:**

This paper presents and describes steps in the testing and reporting process, with examples from a number of countries of barriers encountered in each step, all of which create an undercount of COVID-19 cases. This work raises factors to consider in COVID-19 data and provides recommendations to inform the current situation with COVID-19 as well as issues to be aware of in future pandemics.

## Background

Since the emergence of coronavirus disease 2019 (COVID-19) in Wuhan, China, the world has faced serious data issues, ranging from a lack of transparency on the emergence, spread and nature of the virus to an absence of grounded comparative analyses, with temporal differences considered, about emerging social and economic challenges [1, 2]. Most critically, scientists have lacked data to conduct analyses on non-pharmaceutical interventions (NPIs), including policies and strategies that governments have engaged to mitigate the situation, and how these have varied across regions, presumably affecting both short- and long-term outcomes [1, 2].

Out of all the strategies implemented to date, physical distancing policies have emerged as one of the more effective NPIs to battle COVID-19 [3, 4]. While physical distancing policies have been the mainstay in the battle against COVID-19, there has been a call to understand which forms of physical distancing policies are effective so that targeted and less disruptive measures can be taken in further waves of this pandemic and future pandemics [1, 2, 5, 6]. The best time to institute physical distancing policies and what happens when and how they are eased remain unclear. There are many aspects of distancing, such as recommendations for maintaining a physical distance in public, banning group gatherings (the maximum number and where they take place), or complete lockdowns, that complicate their assessment. Timing and synergies of policies and sociodemographic and political factors play a role in the effectiveness of these policies [7–13]. Some hypothesized sociodemographic factors for increased exposure and severity of COVID-19 include living in a long-term care facility or being institutionalized, age (older), gender (mixed findings), having comorbidities (including high blood pressure, diabetes, obesity, immunocompromised status, tobacco smoking) and social vulnerabilities including race or ethnicity. Also relevant is the carrying capacity and infrastructure of health systems. These factors pose challenges for comparison among countries. Comparison is a prime requisite for evaluating the effectiveness of implementation of various policies between countries. Policymakers and the public have been using metrics such as number of cases, number of deaths and testing capacity to make policy or programme decisions or to decide whether to trust the actions of their governments, respectively.

**Fig. 1:**
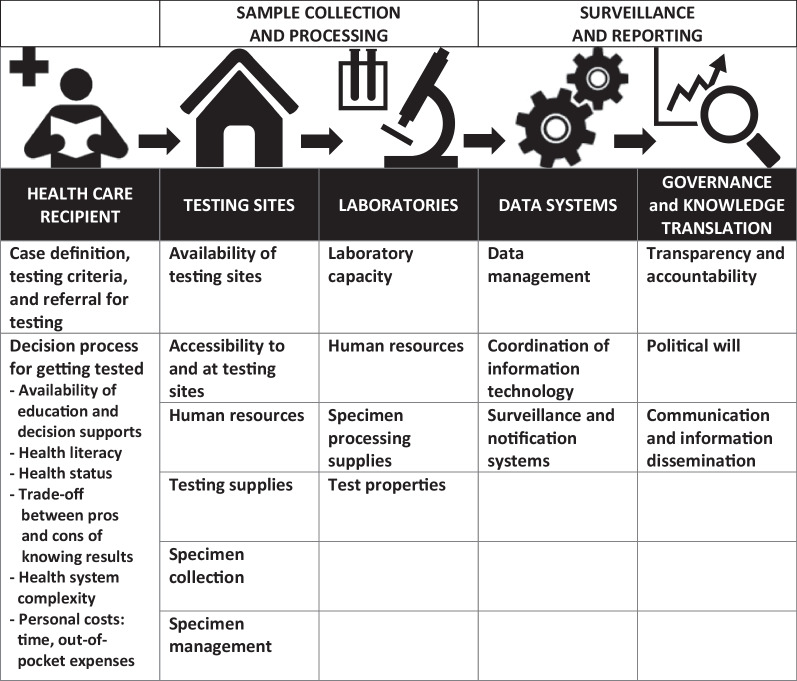
COVID-19 testing and reporting cycle. *The icons in this figure are in the public domain (Creative Commons CC0 1.0 Universal Public Domain) and were obtained from Wikimedia Commons at: https://commons.wikimedia.org/wiki/File:Medical_Library_-_The_Noun_Project.svg; https://commons.wikimedia.org/wiki/File:Home_(85251)_-_The_Noun_Project.svg; https://commons.wikimedia.org/wiki/File:Laboratory_-_The_Noun_Project.svg; https://commons.wikimedia.org/wiki/File:Noun_project_1063.svg; https://commons.wikimedia.org/wiki/File:Analysis_-_The_Noun_Project.svg

An international team of researchers has been collecting data on physical distancing policies and contextual factors, such as health and political systems and demographics, to expedite knowledge translation (which means applying high-quality research evidence to processes of decision making) on the effect of policies and their influence on the epidemiology of COVID-19 [14–16]. Through this work, we identified gaps in the accuracy of reported numbers of COVID-19 cases and deaths, which make cross-country comparisons of the raw data, indexes using the raw data, and policy outcomes challenging [7, 17]. While the work of this team is ongoing, this paper limits the findings from the inception of the pandemic to the end of 2020. It is important to understand the limitations of available COVID-19 data in order to properly inform decision making, especially at the outset as a novel infectious disease. This paper focuses on the testing and reporting cycle (Fig. 1) and provides examples from a number of countries of possible barriers leading to inaccurate data on reported COVID-19 cases. It also describes other cross-cutting implications of COVID-19 data for policy, practice and research, including reported deaths, missing information, implementation of policy, and unpredictable population behaviour. Furthermore, it calls into question analyses performed to date, which do not account for a number of known data gaps.

It is important to note that Fig. 1 only represents the testing and reporting cycle, which leads to counting of cases, and it does not include COVID-19 contact tracing and case management; however, we recognize that testing, contact tracing and case management are intricately linked to each other in the spread of COVID-19 [18, 19]. As ‘Our World in Data’ states, “Without testing there is no data.” [20]. Understanding the links between testing, data and action underlies country responses to the pandemic. Ultimately, this work serves to provide a basis to improve pandemic planning, surveillance and reporting systems, and communications.

In Fig. 1, the first level of testing is at the healthcare recipient level (Sect. “Healthcare recipient level”), followed by sample collection and processing (Sect. “Sample collection and processing”) and surveillance and reporting (Sect. “Surveillance and reporting”). Each level will be further explained below and examples provided as to potential or actual barriers at each level. These descriptions are not exhaustive, and nuanced understanding of the context will be needed to evaluate these steps and potential barriers in different settings.

## Healthcare recipient level

Testing starts with individuals getting tested. There may be times when it is predetermined who gets tested and when, such as health workers getting tested prior to starting work in a long-term care facility or travellers returning from overseas [21, 22]. However, most individuals are tested in the community, where a number of steps predicate individuals’ decisions to seek out testing. First, case definition, testing criteria and referral for testing influence our understanding of what the disease entity is and whether people are encouraged or discouraged to get tested. Given the novel status of COVID-19, there were challenges at the onset of this pandemic in establishing a working case definition. In China, arguably the leader in COVID-19 knowledge at the time, the case definition for reporting changed over time and between places [23]. These definitions were not always consistent with one another. Between 22 January and 12 February 2020, China’s National Health Commission had revised the COVID-19 outbreak response guidelines at least six times, resulting in significant differences in the daily counts due to changes over time in the definition of a case [24]. Adding to the uncertainty, the World Health Organization did not publish case definition guidelines until 16 April 2020, long after many countries had created their own working case definitions [25]. Although changes in methodology are expected as we learn more about the disease and as new variants emerge, these changes have implications for case counts [23, 25]. Yet, communication does need to be flexible during a crisis. For example, little was known about asymptomatic COVID-19 spread at the beginning of the pandemic. As more evidence was garnered on this topic, information about precautions and testing criteria needed to be flexible to keep up with what was known [26].

Not only have case definitions changed over time, criteria for testing have changed over time and across jurisdictions on the basis of a number of factors, such as better understanding of the disease process, availability and capacity for testing, and national and local strategies for addressing the pandemic [27]. In some places, testing criteria were narrow, which discouraged people from getting tested because they did not fit the criteria. In its early response, Canada only tested symptomatic people returning from specific countries known to have high numbers of cases of COVID-19 [18]. Given that there were no treatments and media reported that the hospitals were overwhelmed, people were also discouraged from seeking medical attention unless they warranted hospitalization. If people were feeling unwell, but not needing to be on a ventilator, testing might not have been deemed necessary. Shifting testing criteria and differences in referral channels for testing, such as going through public health or needing a physician referral versus self-referrals, could create additional barriers.

Depending on the testing strategy, whether based on specific criteria or population-based, will make a difference for number of COVID-19 cases identified. Changes in criteria for testing sometimes led to increased demand without a corresponding increase in the availability of testing resources, which then led to delays in accessing tests [28]. Additionally, as different sectors, such as schools, resumed in-person activity, there was an increased demand for testing within certain population groups. Again, testing capacity could not always keep up with demand, leading, in some instances, to further limitations of who could be tested to prioritize resources for testing [27].

In the case that an individual has a choice to get tested, once a person is determined to be eligible for testing, that person has to decide whether or not to get tested, following a decisional process for getting tested, which can be affected by factors such as the availability of education and decision supports, health literacy, health status, trade-offs between knowing their results and potential economic and social consequences, health system complexity, and personal costs, such as time and out-of-pocket expenses [29, 30]. Availability of education and decision support is needed for people to understand that there is a pandemic, what that means, how it might impact them, how and where to get tested (if available) and why getting tested is important for them or their loved ones. This relies on accurate and timely information, which is discussed in more detail in Sect. “Governance and knowledge translation”.

Furthermore, health literacy can involve a general understanding of factors that affect health or it can be specific to a disease entity, such as the virus that causes COVID-19. Health status can decrease the number of people seeking testing if they have mild symptoms and decide it is not worthwhile to seek testing or care, or they may not fit the testing criteria. On the other hand, some people with severe symptoms may not have the physical resources to go to a testing centre.

Of course, even individual-level factors are affected by broader systematic determinants of health. As the gravity of the pandemic took hold, jurisdictions began implementing more robust isolation policies to prevent the spread of COVID-19. These policies included self-isolation or a quarantine period for those who tested positive or who had come in contact with a known case. In many countries, governments provided economic relief to support people who were unable to work [31, 32]. However, in countries such as Brazil and Mexico there were limited social safety nets, and in many other countries such as the USA, COVID-19 exposed gaps in these nets [33, 34]. This created an economic barrier for people to access testing, as a positive test would force them to stay home without adequate financial means to survive. On 28 April 2020, the French Prime Minister, Edouard Philippe, urged the population of France to “protect-test-isolate”; meanwhile, containment measures generated a “disaffiliation process” among migrants and asylum seekers. Absence of work, isolation from French society, and fear of being checked by the police brought individuals into a “disaffiliation zone” marked by social non-existence, in a context of global health crisis [35].

Health systems themselves created a barrier to testing through their slow response to testing requests, causing some individuals to abandon testing [36]. In some countries, testing was expensive and not offered in the poorest communities [37]. For those travelling, mandatory testing, with varying requirements between different countries and potential out-of-pocket costs, increased the complexity of getting tested. Furthermore, competing crises may have lowered the number of people seeking testing due to other, more immediate, priorities, such as floods or wildfires [38–41].

## Sample collection and processing

Once a person decides to seek testing, tests must be available and accessible and there must also be sufficient test processing centres. While these factors are often lumped together, it is important to distinguish these two steps in the testing cycle as they often require different structural and/or operational components.

### Tests and testing sites

For an individual to get tested, there must be availability of testing sites and accessibility to these testing sites. Testing sites may include already available clinic, hospital or community sites, or assessment centres which are created for the purpose of testing. Having separate assessment centres can ease conflicting burdens on already overwhelmed health systems, and they can allow for efficiency in the process of testing and in keeping potentially infectious individuals separate from those who are seen for other ailments. Not only do testing sites have to be available, they have to be accessible. Times of operation, parking and other accessibility considerations are important. Testing sites can be centralized in one or several locations, where people have to find transportation to the sites, or can be mobile sites, which can increase access to those in rural/remote areas or those with mobility or transportation issues. Drive-thru testing has been showcased in countries such as South Korea [42]. However, limitations also exist with drive-thru sites for those who do not own a vehicle, or those who have to drive long distances or endure long wait times [43]. In areas with poor health system infrastructure, lack of access can exacerbate inequities in testing.

Operational components include the need for adequate human resources and testing supplies. In Ontario, Canada, assessment centres were slow to set up and there was a lack of swabs and other testing supplies [18]. In France, laboratories struggled to keep up with testing demands due to delays in receiving chemicals and testing kits produced abroad, given France’s reliance on global supply chains [44]. Bangladesh had a very limited number of case testing capacity in the beginning of the outbreak. The country conducted fewer than 3000 tests in the first four weeks of the outbreak between 8 March and 5 April 2020 for its 164 million population as well as 155,898 overseas passengers, some arriving from hard-hit countries such as Italy, allowing for community transmission [45].

The method of specimen collection and specimen management for processing are also important considerations. Specimen collection has varied between contexts and over time [46]. Nasopharyngeal, nasal and throat swabs have been used in community settings. Saliva tests and blood samples, mainly for hospitalized patients, are other methods of obtaining specimens. Each of these testing modalities has different properties, but none is 100% sensitive or able to pick up all positive cases of COVID-19. There are reports of very ill patients testing negative on multiple occasions on nasopharyngeal samples but subsequently testing positive from lung samples [47, 48]. Specimen management requires the proper labelling, storage and transportation of samples from the testing site to the laboratory for processing.

### Laboratories

Laboratory preparedness and laboratory capacity played crucial roles in COVID-19 testing globally [27, 49]. Issues with this preparedness and capacity, along with lack of testing supplies, resulted in “lack of testing” as a prime factor for not having accurate numbers of COVID-19 cases, especially at the beginning of the pandemic. Laboratory capacity includes human resources and specimen processing supplies, often called the testing kits, which require specific reagents and equipment. Over time, countries with low laboratory preparedness focused on improving their testing capacities [49]. Since the start of the pandemic, Germany was touted as testing widely and therefore having a robust ability to contact trace in order to find people who may transmit the virus causing COVID-19. However, other countries struggled to get testing in place. In the USA, initial tests developed were invalid, which delayed the ability to distribute and complete tests [50]. This was further exacerbated by bureaucratic/institutional red tape which centralized testing to the Centers for Disease Control and Prevention (CDC) and prohibited local public health and commercial laboratories from developing or administering more effective tests [51]. Supply chain management issues for swabs, transport media and reagents slowed down early testing in multiple countries [27].

Once testing methods have been established, there are a number of tests available for COVID-19 [52]. Test properties include the sensitivity and specificity of a test, among others, and these can vary by test. Therefore, the type of test used can also influence case counts. Recent studies have highlighted the need to validate laboratory tests and share the results during a pandemic. Evidence from a study in Alberta, Canada suggested that variations in test sensitivity for the virus causing COVID-19, particularly earlier in a pandemic, can result in “an undercounting of cases by nearly a factor of two” (p. 398) [53]. With rapid tests and home-approved testing kits available during the course of the pandemic, testing properties can vary even more greatly [52, 54, 55].

## Surveillance and reporting

Once individuals have been tested and the results are processed, surveillance and reporting systems must be in place to communicate that information back to individuals, public health officials or others involved in case management or treatment, and to politicians and other stakeholders to act on this information and prevent further spread.

### Data systems

Data management refers to the inputting and tracking of data. However, because of the need to quickly and accurately inform the public and decision makers in the time of a crisis, coordination of information technology is needed to align all the various data management systems within a jurisdiction and internationally. For example, each hospital system, clinic or laboratory may have separate electronic medical record or data management systems. Not many countries maintain a common database system for COVID-19-related management (testing, response, etc.). Even if database management systems are in place, lack of trained professionals, serious lags in updating data, challenges with interdepartmental coordination among various task force members, and new innovations such as artificial intelligence, health tracking apps, telemedicine and big data, which are suddenly in place, can lead to disrupted transparency. An exception is China, which developed a highly responsive national notifiable disease reporting system (NNDRS) in the aftermath of severe acute respiratory syndrome (SARS) [56, 57]. The United Nations Department of Economic and Social Affairs statistics division launched a common website for improving the data capacities of countries [58]. This information has to be further coordinated to create larger and more robust surveillance and notification systems. Robust surveillance systems help decision makers know what is happening locally or how a disease is moving through populations. Notification systems are needed for sharing information between the testing site, laboratory and public health or local health agencies for case management and contact tracing and for letting people know their test results in a timely manner to help prevent further spread. The COVID Tracking Project has highlighted many discrepancies in USA reporting and surveillance, demonstrating unreliability of the data [59]. For example, hospitals were required to change how COVID-19 data were relayed to the federal government, and the switch from reporting through the CDC to the Health and Human Services (HHS) system resulted in misreporting of data and administrative lags across several states. Countries’ national-level CDCs collect information from state and local sources. The time lag can hence be one of the reasons for misleading the overall comprehensive pandemic impact. Lastly, with rapid, point-of-care and home tests available, keeping track of positive cases may be even more difficult, and COVID-19 case counts could be even further artificially decreased [60]. These tests could make contact tracing even more difficult if there is a lack of disclosure from the user end. It is important to note that, while there are many available sites for international COVID-19 data comparisons, including John’s Hopkins COVID-19 Dashboard [61], Worldometer [62], Our World in Data [20] and the World Health Organization (WHO) COVID-19 Dashboard [63], these all rely on locally-acquired data for their reporting, and therefore fall into and potentially augment the same fallacies discussed in this paper.

### Governance and knowledge translation

Even with robust surveillance and notification systems, transparency and accountability are important for informing decision makers and the public. Decision makers need to know what the health and laboratory systems are finding so that evidence-informed policy and practice decisions can be made for the public good. At the same time, trust in government and government responses rely in part on perceived transparency of government by the public [64, 65]. Accountability spans all through the spectrum discussed in the testing and reporting cycle, in a whole-of-society approach. Individuals are accountable for knowing when to get tested, getting tested and following public health guidelines and other policies. The public health and healthcare systems are accountable for planning testing and sharing information. Decision makers are accountable for transparency in sharing information, communicating appropriately with the public and relevant stakeholders, and making decisions for those they represent. In parts of Russia, there were two separate reports for those who died from COVID-19 and those who were positive but died from other causes [25]. In Florida, state officials instructed medical examiners to remove causes of death in their lists [66]. In China, despite having a highly responsive national data surveillance and reporting system, at the beginning of the pandemic, cases were only reported to the system once they had been approved by local members of government who only allowed cases with a direct connection to the original source of the outbreak, the seafood market, to be recorded [67].

Political will has been shown to be a barrier or facilitator in the fight against COVID-19. Examples of good leadership and political will can be found in places like New Zealand, where decisions were made early on, implemented, supported and continued to be informed by emerging evidence, or as described, following “science and empathy”[68]. Poor leadership has also come through clearly during this pandemic. Tanzania, Iran, the USA, Brazil and Egypt are only a handful of countries demonstrating the impact of political will on the course of the pandemic, in some cases resulting from subversion and corruption. Communication in these countries was often not transparent or mixed, and accountability for the lack of decision making or poor decision making was limited or non-existent in the pandemic’s outset. Tanzania stopped reporting cases due to political optics [30, 69]. Iran’s Health Ministry reported 14,405 deaths due to COVID-19 through July 2020, which was a significant discrepancy from the 42,000 deaths recorded through government records [70]. The number of cases was also almost double those reported, 451,024 as compared with 278,827. One main reason for releasing underestimated information about the cases was considered to be upcoming parliamentary elections [70, 71]. The former president of the USA, Donald Trump, often flouted public health and healthcare expert advice [72]. The Washington Post reported that Brazil was testing 12 times fewer people than Iran and 32 times fewer people than the USA, and hospitalized patients and some healthcare professionals were not tested in an effort to lower the case numbers [73]. Hiding numbers of deaths from COVID-19, whether intentionally or inadvertently, shored up far-right supporters of Brazil’s President Bolsonaro at a time when he was facing possible charges of impeachment for corruption and helped bolster the President’s messaging that the pandemic was under control. This further enabled a large swath of the population to call for less strict rules around COVID-19 and a quick reopening of the economy. Similarly, in July 2020, it was reported that at least eight doctors and six journalists had been arrested because they criticized the Egyptian government’s response to the pandemic [74].

Lastly, communication and information dissemination link to every piece of this process. Why, when and how people seek testing, how and where to set up testing sites, supply chain management, setting up and managing data systems, and policy decision making all work in a cycle. Good communication between systems and dissemination of information to the public and relevant stakeholders is imperative during a crisis, such as the COVID-19 pandemic. The amount of information available and rapid change in information creates an infodemic problem. ‘Infodemic’ is a term used by the WHO in the context of COVID-19 and refers to informational problems, such as misinformation and fake news, that accompany the pandemic [75]. Addressing the infodemic issue was highlighted as one of the prominent factors needed to improve future global mitigation efforts [76]. A report published in the second week of April 2020 by the Reuters Institute for the Study of Journalism at the University of Oxford found that roughly one-third of social media users across the USA, as well as Argentina, Germany, South Korea, Spain and the UK, reported seeing false or misleading information about COVID-19 [77]. The presidents of Brazil and the USA were themselves sources of misinformation, as they were seen in public without masks and touting the benefits of hydroxychloroquine after it was largely known that harms outweighed benefits of its use [72, 78, 79]. Having clear public health communications, from trusted sources, and breaking down silos between systems could be helpful in combating ever-changing information during a pandemic.

## Other implications of COVID-19 data for policy, practice and research

There are several cross-cutting issues separate, but related, to the testing and reporting cycle which arose during this work. These issues also affect COVID-19 case counts and optimal timing of policies: how deaths are reported, missing information, implementation of policies, and unpredictable population behaviour.

### Reported deaths

Deaths from COVID-19 tend to occur weeks after infection; therefore, assessments of policy changes using death counts need to account for this timing. However, reported death counts from COVID-19 carry many similar limitations given lack of testing for those who are deceased, attributing cause of death to COVID-19-related complications, processes for declaring deaths and causes of deaths, and lack of transparency [80]. In Brazil, hospitalized patients were not being tested, and deaths were attributed to respiratory ailments [73]. Further, COVID-19 deaths from the City of Rio de Janeiro’s dashboard were blacked out for 4 days in May (22–26 May 2020) [81]. When the dashboard was restarted, the death count was artificially lowered by changing the cause of death from COVID-19 to its comorbidities. Additional changes included requiring a confirmed COVID-19 test at the time of death in order for the death certificate to list COVID-19 as the cause; however, the results of the test often came after the death certificates were issued [81]. In Italy, the reverse occurred where only those in hospital were counted as COVID-19 deaths, while many people died at home or in care homes without being tested [82, 83]. In Ireland, early discrepancies in reported deaths were noted between official government figures and an increase in deaths noted on the website Rip.ie, which has served as a public forum disclosing deaths and wake information in line with Irish funeral traditions. Information from this forum was used to re-assess mortality and in some cases aid epidemiological modelling [84].

### Missing information

Given the lack of access to treatments at the beginning of the pandemic, understanding who was at highest risk of obtaining or dying from COVID-19 was important to know in order to develop appropriate policies that balanced health with social and economic impacts of the pandemic. Early data showed a sex and age gradient for COVID-19 cases and deaths. However, not all countries report data by sex and/or age. Race/ethnicity and sociodemographic findings were not collected or reported early in the pandemic [85]. France has been criticized for laws which prohibit the collection of race and ethnicity data, since they lack data which demonstrate whether certain groups are overrepresented in COVID-19 cases and deaths [86, 87]. Another aspect of missing data early in the pandemic was that of asymptomatic spread. Due to limited testing early in the pandemic, asymptomatic cases were not picked up. Population-based studies are being conducted to better understand the role of asymptomatic and pre-symptomatic spread of COVID-19 in different population groups, such as children [26].

### Implementation of policy

Population-level strategies since the start of the pandemic and reported findings in the literature go hand and hand. Cause and effect are difficult to attribute. For example, early literature looking at the role of children on the spread of COVID-19 found that children played a small role. This was to be expected given that many schools around the world closed, and children would not be exposed through transportation and workplaces as adults would be. Therefore, family spread would naturally flow from adults to children given these circumstances. In addition, many places were not testing mild to asymptomatic cases, which were more commonly found in children. Publications early on related to the few severe COVID-19 cases in children or to school-related cases in places that had low community transmission rates of COVID-19 and were following public health guidelines [88]. Limitations of these data have been described, yet findings have been used to justify specific policies in places that were dissimilar, with expected results ensuing, such as an increase in community transmission and school closures due to COVID-19 infections [89]. Therefore, it is even more important to understand the context of policies before applying them to various jurisdictions.

### Unpredictable population behaviour

There is a difference between stated policy, implementation and enforcement. To understand which policies worked to combat COVID-19, it is important to consider the level of compliance with stated policies. Some people may follow recommended approaches for protective actions while others may not comply and see these recommendations as problematic [90]. For example, people may change their behaviours in anticipation of an announced change; for example, individuals may start working from home even before it is enforced or if it is never officially mandated, or people may go on a shopping spree prior to known closures [91, 92]. Of course, people’s behaviours may also be dependent on a disconnect between policy messages at different levels of government and exacerbated by rapid updates in a fast-moving pandemic of unknown properties and the associated information overload. Therefore, communication management and clarity are of utmost importance during a crisis.

## Discussion and recommendations

The need for cross-country comparison is necessary for understanding the effectiveness of policies in various countries. Policy decisions are being made and judged on the basis of case numbers, deaths and testing, among others. Understanding the steps and barriers in testing and reporting data related to COVID-19 case numbers can help address the limitations of data to strengthen these systems for future pandemics and can also help in the interpretation of findings across jurisdictions. Robust and timely public health measures are needed to decrease the health, social and economic ramifications of the pandemic. Even with available vaccines, it will still take time to have sufficient population coverage internationally.

There are a few assumptions considered in this paper. First, we assume that the reported numbers for each country are not inflated. There could be some cases that are counted more than once if repeated tests are taken and the person continues to test positive. Most data do not disclose how often this occurs, but it is likely not a significant issue for population reports, at least at from the beginning of the pandemic [20]. Next, ideally COVID-19 case counts are accurate. This is the assumption that is made by policymakers and the public in judging their decisions and their outcomes. We argue that the reported COVID-19 data are likely an undercount of actual cases. The reasons are highlighted in this paper.

Future global discussions will continue around who is most affected by COVID-19 and how to best prepare for pandemics, among others. COVID-19 case and death counts will be used in determining successful approaches. It is important to understand the context of COVID-19 data in these discussions, especially with respect to other global indicators that may look to COVID-19 data, such as the Sustainable Development Goals (SDGs) through improvement of early warning, risk reduction and management of national and global health risks [93]. Specifically, SDG 3 (good health and wellbeing) with an emphasis on highlighting the lacunas in informed data tying policy and epidemiology, SDG 10 to reduce inequalities within and among countries, and SDG 16 (peace, justice and strong institutions) with a goal to build effective and accountable institutions at all levels. This research also contributes to the Sendai Framework for Disaster Risk Reduction, specifically priority 2, strengthening disaster risk governance to manage disaster risk, and priority 3, investing in disaster risk reduction for resilience [94]. Unfortunately, there is little published on good governance in reporting systems during COVID-19, and our findings in this area are limited to media and news sources. Future research could focus on this critical aspect.

Decision makers could consider the following overarching recommendations, contextualized to their individual jurisdictions (i.e. regional, country, province, territory, state), to evaluate the testing and reporting cycle and improve accuracy and comparability of COVID-19 data:

1. *Understand barriers to accurate testing and reporting*—This paper lays out the steps in the testing and reporting process and components of these steps. Barriers are described at each of these steps, and examples are provided.
2. *Address barriers to testing and reporting*—Understanding barriers in the testing and reporting process can uncover facilitators. Each setting will deal with different barriers. Ultimately, political will, capacity building and robust information systems will be needed to address any of these barriers.
3. *Transparency and accountability for surveillance and reporting*—Any attempt to assign causality to these policies must take into account the timing and quality of surveillance data. Data quality issues, such as completeness, accuracy, timeliness, reliability, relevance and consistency, are important for surveillance and reporting [95, 96].
4. *Invest in health system strengthening, including surveillance and all-hazards emergency response plans*—COVID-19, as this and past pandemics have shown, is not just a health issue, and instead requires community, health systems, social systems and policy approaches to mitigate its effects. Preparing for infectious disease outbreaks and other crises needs to incorporate all-hazards emergency response plans in order to have all the necessary resources in place at the time of the events.
5. *Identify promising communication strategies*—Research is needed to understand how messages conveyed at all stages of a pandemic are received and understood at the micro-level and used by the public [97]. Development of communication strategies aimed at promoting good understanding of information may defer inappropriate behaviours.
6. *Invest in research to further understand data reporting systems and policy strategies and implementation*. Research could compare global COVID-19 data reporting platforms mentioned in this article to see from where they obtained their raw data to further understand data reporting accuracy and comparability of data over time and whether any limitations of data were noted. Further research could address what policy and implementation strategies worked in a variety of settings to strengthen future recommendations for emerging pandemics.

## Conclusion

The use and effectiveness of government responses, specifically pertaining to physical distancing policies in the COVID-19 pandemic, has been evolving constantly. Testing is a measure of response performance and becomes a focal point during an infectious disease pandemic as all countries are faced with a similar situation. COVID-19 represents a unique opportunity to evaluate and measure success by countries to control its spread and address social and economic impacts of interventions. Understanding limitations of COVID-19 case counts by addressing factors related to testing and reporting will strengthen country responses to this and future pandemics and increase the reliability of knowledge gained by cross-country comparisons. Alarmingly, with COVID-19 having asymptomatic spread, lack of testing can discredit the efforts of an entire community, not to say an entire population.

## Data Availability

All data generated or analysed during this study are included in this published article.

## Abbreviations

CDC: Centers for Disease Control and Prevention
COVID-19: Coronavirus disease 2019
HHS: Health and Human Services
NNDRS: National notifiable disease reporting system
NPIs: Non-pharmaceutical interventions
SARS: Severe acute respiratory syndrome
SDGs: Sustainable Development Goals
WHO: World Health Organization
